# Supplementary material for: Highly indistinguishable and strongly entangled photons from symmetric GaAs quantum dots
Source: Nat Commun. 2017 May 26;8:15506. doi: 10.1038/ncomms15506 (PMC5458553; doi:10.1038/ncomms15506)
Supplement: Supplementary Information — Supplementary Notes, Supplementary Figures, Supplementary Tables and Supplementary References [file ncomms15506-s1.pdf]

Supplementary Note 1: Calculation of the two-photon interference visibility

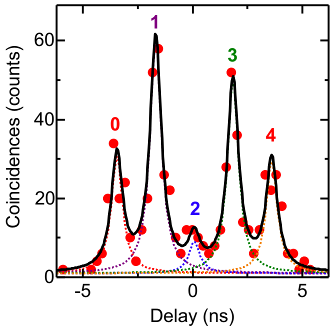

Supplementary Figure 1 **Evaluation of the visibility of two-photon interference.** Two-photon interference measurement of a representative quantum dot. The dashed coloured lines are the fits of peak 0 (red), 1 (violet), 2 (blue), 3 (green) and 4 (orange), respectively. The black line is the sum of all single peak fits. The areas under the peaks 1, 2 and 3 are used for the calculations of the two-photon interference visibility.

In order to extract the visibility of two-photon interference, we fit the experimental data using the following equation:

$$f(t) = y_0 + \sum_{i=0}^{4} \frac{2A_i w}{\pi \cdot (w^2 + 4(x - (x_0 + i \cdot d)))} \tag{1}$$

where y_0 is the offset, A_i the area of peak i, x_0 the position of the first peak, w the width of the peaks and d the temporal distance between the peaks. From the experimental conditions we expect that the distance d between the peaks is the same. Furthermore, all the peaks should have the same width, which is mainly determined by the time jitter of the avalanche photodiode (500 ps). In general, one would also expect that peak 0 and 4 as well as 1 and 3 are equal in intensity. However, we have to consider the slightly different intensity between the two excitation pulses as well as the different detection efficiency for both fibre outputs. This is taken into account by leaving the A_i as free parameters.

We have then calculated the two-photon interference visibility via

$$V_{\mathrm{TPE}} = 1 - \frac{2 \cdot A_2}{A_1 + A_3}. \tag{2}$$

To correct for the imperfections of the beam splitter we measured the mode overlap $(1-\varepsilon) = 0.96 \pm 0.01$ (using the fibre beam splitter to perform a Michelson measurement on the shaped excitation laser), the transmission coefficient $T = 0.48 \pm 0.005$ and the reflection coefficient $R = 0.52 \pm 0.005$ (using a power meter) and calculate the corrected visibility with (see Supplementary information of[1] and[2]):

$$V_{\mathrm{TPEcor}} = \frac{1}{(1-\varepsilon)^2} \left(2g^2(0) + \frac{R^2+T^2}{2RT} - \frac{A_2}{A_1+A_3} (2 + g^2(0) \frac{(R^2+T^2)}{RT}) \right), \tag{3}$$

where we put $g^2(0) = 0$.

The uncorrected as well as the corrected values are listed in Supplementary Table 1.

QD	visibility X	visibility XX	corrected visibility X	corrected visibility XX
1	0.79 ± 0.07	0.85 ± 0.05	0.86 ± 0.09	0.93 ± 0.07
2	0.68 ± 0.04	0.67 ± 0.06	0.74 ± 0.06	0.73 ± 0.07
3	0.63 ± 0.05	0.70 ± 0.05	0.69 ± 0.06	0.76 ± 0.06

Supplementary Table 1 **Results of the two-photon interference experiment.** Calculated two-photon interference visibility directly from the fitted peak areas for quantum dot QD1-QD3 for exciton (X) and biexciton (XX). The corrected visibility accounts for the imperfections of the fibre beam splitter. The errors are calculated assuming a Poisson statistics for the correlation counts and Gaussian error propagation.

Supplementary Note 2: Two-photon interference visibility at longer pulse separation

To prove the behaviour of the photon indistinguishability under a longer pulse separation, i.e., a longer time separation between the creation of the two interfering photons, we performed a comparison of the visibility of biexciton (XX) photons from an arbitrarily chosen quantum dot (QD) at a pulse separation of 2 ns and 12.5 ns. The measurement data are plotted in Supplementary Figure 2. The visibility drops from 0.62 ± 0.06 to 0.43 ± 0.09 by increasing the pulse separation. This is a known problem[3] in the literature and is related to spectral diffusion due to random charge fluctuations in the sample.

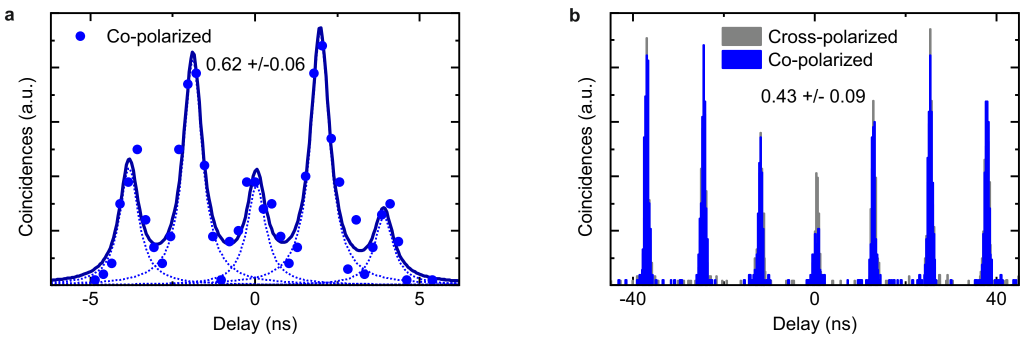

Supplementary Figure 2 **Two-photon interference visibility at different pulse separations.** (a) Two-photon interference measurement of an biexciton emission line of representative quantum dot at an excitation pulse separation of 2 ns in co-polarized configuration. (b) Same measurement as before, but with a pulse separation of 12.5 ns in co- (blue) and cross-polarized (grey) configuration. The corrected values of the visibility are reported in the corresponding figures.

Supplementary Note 3: Decay time and coherence time measurements

We measured the decay time of exciton (X) and XX under two-photon excitation using a detector with a time resolution of around 50 ps. The acquired data are presented in Supplementary Figure 3 (a). After a deconvolution with the system response function (see inset of Supplementary Figure 3 (a)) the decay time T_1 of exciton (X) and XX are found to be 256±5 ps and 129±4 ps, respectively.

The coherence times of X and XX for the same QD have been measured with a Michelson interferometer. The acquired data in Supplementary Figure 3 (b) are fitted with an exponential decay function:

$$V(t) = y_0 + A \cdot e^{-t/T_2} \tag{4}$$

where V is the interference fringe visibility, A the amplitude, y_0 the offset and T_2 is the decay constant. The parameters y_0 and A, which under ideal experimental conditions should be 0 and 1 are left as free parameters because of small imperfections of the mode overlap. The calculated coherence time T_2 for X and XX are 305±39 ps and 109±15 ps, respectively. From the lifetime and coherence-times we can obtain an estimate for the visibility of two-photon interference by using (see[4]):

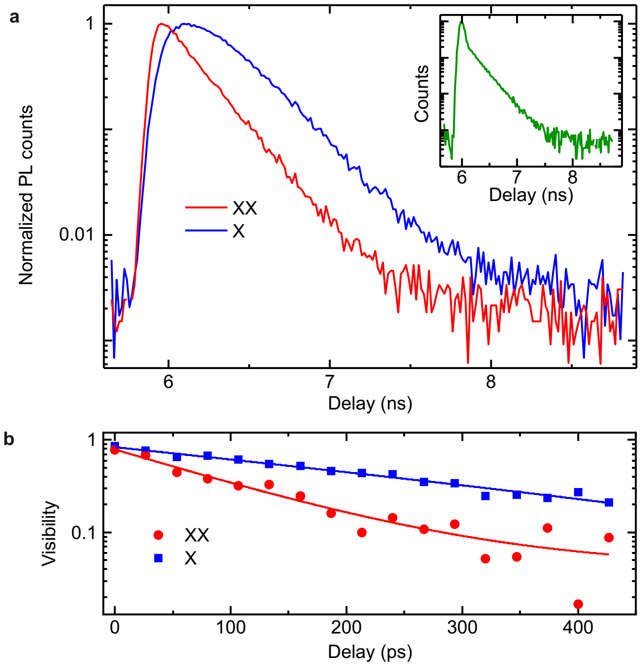

Supplementary Figure 3 **Measurement of the decay time and coherence time of quantum dot QD3.** (a) Decay time measurements of QD3 for excition (X) (see blue line) and biexciton (XX) (see red line), respectively. The inset shows the instrument response function of the detector. (b) Coherence time measurement of QD3 for X (blue) and XX (red), respectively. The solid lines are fits to the data using exponential decay functions.

$$V_{\mathrm{TPE}} = \frac{T_2}{2T_1}, \tag{5}$$

which is found to be $V_{\mathrm{TPE}} = 0.6$ and $V_{\mathrm{TPE}} = 0.4$ for X and XX, respectively. The data from the two-photon interference experiment (see Supplementary Table 1), yields a higher visibility for the same QD ($V_{\mathrm{TPE}} = 0.69$ and $V_{\mathrm{TPE}} = 0.76$ for X and XX, respectively). This discrepancy can be explained by the presence of decoherence processes on a time scale that is larger than the time delay between the two laser pulses used to generate the interfering photons. A closer inspection of the difference between the determined values indicates that the effect on the XX is more dominant. A possible explanation is that the XX is more sensitive to spectral diffusion mediated by temporally charged defects than the X state, but additional investigations (see for example[5]) are needed to test this hypothesis.

Supplementary Note 4: Source of entanglement degradation

Although a fidelity of 0.94 is high, it is not yet perfect. We have investigated this in more detail by using a simple model according to[6]. The density matrix of the model system in the $[H_{\mathrm{XX}}H_{\mathrm{X}}, H_{\mathrm{XX}}V_{\mathrm{X}}, V_{\mathrm{XX}}H_{\mathrm{X}}, V_{\mathrm{XX}}V_{\mathrm{X}}]$ basis is given by:

$$\rho = 1/4 \begin{pmatrix} 1+kg'_{\mathrm{H,V}} & 0 & 0 & 2kg_{\mathrm{H,V}}z^* \\ 0 & 1-kg'_{\mathrm{H,V}} & 0 & 0 \\ 0 & 0 & 1-kg'_{\mathrm{H,V}} & 0 \\ 2kg_{\mathrm{H,V}}z & 0 & 0 & 1+kg'_{\mathrm{H,V}} \end{pmatrix} \tag{6}$$

where $g'_{\mathrm{H,V}} = 1/(1+T_1/T_{\mathrm{SS}})$, $g_{\mathrm{H,V}} = 1/(1+T_1/T_{\mathrm{SS}}+T_1/T_{\mathrm{HV}})$, $z = \frac{1+ix}{1+x^2}$, $x = \frac{g_{\mathrm{H,V}}sT_1}{\hbar}$ with T_{SS} the spin scattering time, T_{HV} the cross dephasing, T_1 the exciton decay time, the fine structure splitting (FSS) s and the fraction k of photons emitted exclusively by the QD. From this density matrix it is possible to estimate the entanglement fidelity, which reads:

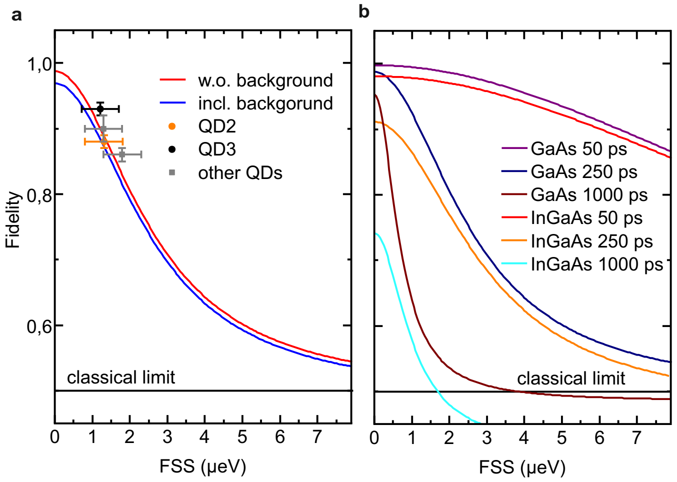

Supplementary Figure 4 **Entanglement fidelity versus fine structure splitting**. (a) The solid curve is calculated with (blue) and without (red) background illumination, respectively, for GaAs quantum dots (QDs). The orange and black dot represent the measured fidelity of quantum dot QD2 and QD3, respectively, while gray rectangles are values obtained by two additional QDs not mentioned in the main text. The error bars in the abscissa are calculated by assuming a Poisson statistics for the correlation counts and Gaussian error propagation. The error of the fine structure splitting (FSS) is estimated by the fitting of polarization resolved measurements. The black line is the limit for classical correlation (fidelity< 0.5) between the exciton and and biexciton photons. (b) Calculated fidelity versus FSS for different exciton decay times (50 ps, 250 ps and 1000 ps) for GaAs and InGaAs QDs.

$$f = \frac{1}{4}(1 + kg'_{\mathrm{H,V}} + \frac{2kg'_{\mathrm{H,V}}}{1+x^2}) \quad (7)$$

Using the measured lifetime of the exciton transition for T_1, the value of the $g^{(2)}(0)$ to estimate k, and considering that the effect of cross-dephasing can be safely neglected[6], i.e., $g'_{\mathrm{H,V}} = g_{\mathrm{H,V}}$, the only unknown parameter entering in Supplementary Equation 7 is the spin-scattering time. Here, we assume that T_{SS} is mainly determined by the Fermi-contact interaction between the confined electron and the nuclear spins[7], while the heavy-hole dephasing related to a dipole-dipole interaction[7,8], is assumed to be weaker and is therefore not considered. Taking the values from the literature ($T_{\mathrm{SS}} = 15$ ns for GaAs QDs[9]), we can therefore estimate the behaviour of the entanglement fidelity as a function of the FSS. This is shown in Supplementary Figure 4 (a) with and without including the effect of the background laser (as estimated by the value of the auto-correlation function for X, that is, $k = 1 - g^{(2)}(0) = 0.975$ (see Supplementary Figure 5)). The obtained experimental data are in consensus with the theoretical curve and support the supposition that the reduced value of fidelity for QD2 is due to to the background of laser photons. Most importantly, this figure highlights that near-unity values (0.99) of entanglement fidelity can be obtained in QDs with suppressed FSS (s=0). This is in contrast to what is reported for InGaAs QDs where maximum values of around 0.9 were predicted[10]. It is therefore interesting to compare directly the two systems using the very same model. Supplementary Figure 4 (b) shows the results of such a comparison, as obtained by using the literature values for the spin scattering times ($T_{\mathrm{SS}} = 1.9$ ns for InGaAs[11]) and by assuming identical lifetimes. The calculations indeed confirm that the maximum entanglement fidelity that can be reached in InGaAs QDs is roughly 10% lower than in GaAs QDs, that is, bound to values around 90%. Moreover, the calculations also highlight the importance of having short X lifetimes to reach high values of entanglement at non-zero FSS, although a combination of FSS=0 and short X lifetime is the key to reach the ideal levels of entanglement needed by the envisioned applications. From this perspective, another potential source of technical problems is the rejection of the stray light. As the laser is spectrally separated from the X and XX line a fibre Bragg grating could be used to filter out the laser emission[12]. Alternatively, rejection of the straight light can be achieved by decoupling excitation and collection. This solution – which has been already employed in[13] and[14] – can also be used in combination with different concepts for on-chip quantum optics[15].

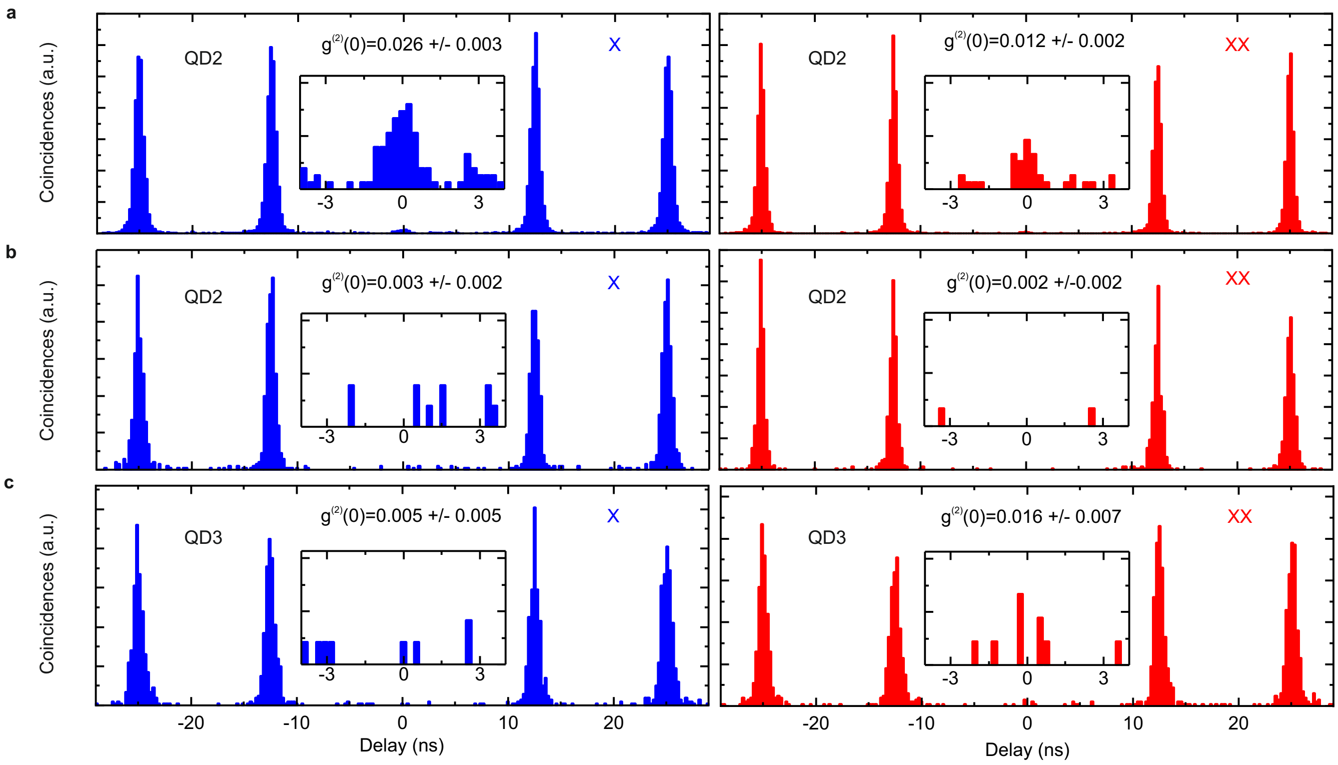

Supplementary Figure 5 **Auto correlation function measurements of quantum dot QD1 and QD3.** (a) Measurements of QD2 without polarization suppression of the laser for the exciton (X) and biexciton (XX) emission line. The measurement gives a value of $g^{(2)}(0) = 0.025 \pm 0.004$ and $g^{(2)}(0) = 0.012 \pm 0.002$ for X and XX, respectively. (b) By inserting in the microphotoluminescence path a polarizer in cross-configurations with respect to the excitation laser (polarization suppression), it turns out that a value of $\mathrm{g}^{(2)}(0) < 0.002 \pm 0.002$ is achievable. This indicates that the non-zero value of the $g^{(2)}(0)$ is not a property of the QD, but an artifact of the experimental setup, which lowers the degree of entanglement that we measure, as polarization suppression cannot be used in quantum state tomography. (c) With QD3 we took special care in rejecting the scattered light from the laser and, without using polarization suppression we measure $g^{(2)}(0) = 0.005 \pm 0.005$ and $g^{(2)}(0) = 0.016 \pm 0.007$ for X and XX, respectively. The insets show the correlation around delay 0 in more detail.

Supplementary Note 5: Evaluation of the entanglement fidelity

For calculating the fidelity from the 6 cross-correlation measurements reported in Fig. 3(b) and (c) of the main text, the raw counts at $g^{(2)}(0)$ within a time window of 1.6 ns (the bunching peak is within this time window) are summed up for all polarization settings. The degree of correlations is calculated via:

$$C_\mu = \frac{g^{(2)}_{\mathrm{XX,X}} - g^{(2)}_{\mathrm{XX,\overline{X}}}}{g^{(2)}_{\mathrm{XX,X}} + g^{(2)}_{\mathrm{XX,\overline{X}}}}, \tag{8}$$

where $g^{(2)}_{\mathrm{XX,X}}$ is the co- and $g^{(2)}_{\mathrm{XX,\overline{X}}}$ the cross-polarized correlation measurement, respectively, in the base μ (linear, diagonal and circular). In Supplementary Table 2 the correlations for QD2 and QD3 are shown.

μ	QD2	QD3
linear	0.95 ± 0.01	0.91 ± 0.01
diagonal	0.71 ± 0.01	0.96 ± 0.01
circular	-0.84 ± 0.02	-0.87 ± 0.02

Supplementary Table 2 **Correlations for the different polarization bases μ for quantum dot QD2 and QD3.**

The fidelity is given by[6]:

$$f = \frac{1 + C_{\mathrm{linear}} + C_{\mathrm{diagonal}} - C_{\mathrm{circular}}}{4}, \tag{9}$$

which yields $f = 0.88 \pm 0.01$ and $f = 0.94 \pm 0.01$ for QD2 and QD3, respectively. The errors are calculated by assuming a Poisson distribution for the correlation counts and propagated by Gaussian error propagation.

Supplementary References

1. Somaschi, N. *et al.* Near-optimal single-photon sources in the solid state. *Nature Photonics* **10**, 340–345 (2016).
2. Giesz, V. *et al.* Cavity-enhanced two-photon interference using remote quantum dot sources. *Phys. Rev. B* **92**, 161302 (2015).
3. Thoma, A. *et al.* Exploring Dephasing of a Solid-State Quantum Emitter via Time- and Temperature-Dependent Hong-Ou-Mandel Experiments. *Phys. Rev. Lett.* **116**, 033601 (2016).
4. Gold, P. *et al.* Two-photon interference from remote quantum dots with inhomogeneously broadened linewidths. *Phys. Rev. B* **89**, 035313 (2014).
5. Kuhlmann, A. V. *et al.* Charge noise and spin noise in a semiconductor quantum device. *Nature Physics* **9**, 570–575 (2013).
6. Hudson, A. J. *et al.* Coherence of an entangled exciton-photon state. *Phys. Rev. Lett.* **99**, 266802 (2007).
7. Urbaszek, B. *et al.* Nuclear spin physics in quantum dots: An optical investigation. *Rev. Mod. Phys.* **85**, 79–133 (2013).
8. Chekhovich, E. A. *et al.* Isotope sensitive measurement of the hole-nuclear spin interaction in quantum dots. *Nature Physics* **9**, 1–5 (2011).
9. Chekhovich, E. A. *et al.* Nuclear spin effects in semiconductor quantum dots. *Nature Materials* **12**, 694–504 (2013).
10. Stevenson, R. M. *et al.* Coherent entangled light generated by quantum dots in the presence of nuclear magnetic fields. *Preprint at: arXiv:1103.2969* (2011).
11. Stockill, R. *et al.* Quantum dot spin coherence governed by a strained nuclear environment. *Nature Communications* **7**, 12745 (2016).
12. Muñoz-Matutano, G. *et al.* All-Optical Fiber Hanbury Brown and Twiss Interferometer to study 1300nm single photon emission of a metamorphic InAs Quantum Dot. *Scientific Reports* **6**, 27214 (2016).
13. Müller, M., Bounouar, S., Jöns, K. D., Glässl, M. & Michler, P. A semiconductor source of triggered entangled photon pairs. *Nature Photonics* **8**, 224–228 (2014).
14. Jayakumar, H. *et al.* Time-bin entangled photons from a quantum dot. *Nature Communications* **5**, 4251 (2014).
15. Stock, E. *et al.* On-Chip Quantum Optics with Quantum Dot Microcavities. *Advance Materials* **25**, 707–710 (2012).
